# Resource Recovery of Spent Lithium-Ion Battery Cathode Materials by a Supercritical Carbon Dioxide System

**DOI:** 10.3390/molecules29071638

**Published:** 2024-04-05

**Authors:** Yuanpeng Fu, Xianshu Dong, Burçak Ebin

**Affiliations:** 1 School of Mining Engineering, Taiyuan University of Technology, Taiyuan 030024, China; fuyuanpeng@tyut.edu.cn; 2 Key Laboratory of Coal Processing and Efficient Utilization, China University of Mining and Technology, Ministry of Education, Xuzhou 221116, China; 3 Department of Chemistry and Chemical Engineering, Nuclear Chemistry and Industrial Material Recycling, Chalmers University of Technology, 412 96 Gothenburg, Sweden

**Keywords:** lithium-ion batteries, cathode material, supercritical CO_2_, dimethyl sulfoxide, liberation

## Abstract

The increasing global market size of high-energy storage devices due to the boom in electric vehicles and portable electronics has caused the battery industry to produce a lot of waste lithium-ion batteries. The liberation and de-agglomeration of cathode material are the necessary procedures to improve the recycling derived from spent lithium-ion batteries, as well as enabling the direct recycling pathway. In this study, the supercritical (SC) CO_2_ was innovatively adapted to enable the recycling of spent lithium-ion batteries (LIBs) based on facilitating the interaction with a binder and dimethyl sulfoxide (DMSO) co-solvent. The results show that the optimum experimental conditions to liberate the cathode particles are processing at a temperature of 70 °C and 80 bar pressure for a duration of 20 min. During the treatment, polyvinylidene fluoride (PVDF) was dissolved in the SC fluid system and collected in the dimethyl sulfoxide (DMSO), as detected by the Fourier Transform Infrared Spectrometer (FTIR). The liberation yield of the cathode from the current collector reaches 96.7% under optimal conditions and thus, the cathode particles are dispersed into smaller fragments. Afterwards, PVDF can be precipitated and reused. In addition, there is no hydrogen fluoride (HF) gas emission due to binder decomposition in the suggested process. The proposed SC-CO_2_ and co-solvent system effectively separate the PVDF from Li-ion battery electrodes. Thus, this approach is promising as an alternative pre-treatment method due to its efficiency, relatively low energy consumption, and environmental benign features.

## 1. Introduction

Lithium-ion batteries (LIBs), as a typical power source, have been widely used in consumer electronics, electric vehicles (EVs), and the energy storage field since their commercial application in the 1990s [1,2,3], which is due to their desirable electrochemical properties in terms of long service life, excellent circulation performance, high energy density, etc. [4,5,6]. The latest data has shown that global production reached more than 957.7 GWh in 2022, which can be attributed to the increasing trend in EVs [7,8]. However, the consequence of such increased usage will be the disposal of a large number of spent batteries. The inappropriate disposal of spent LIBs can pose a serious threat to the environment due to their hazardous constituents, such as heavy metals and electrolytes [9,10]. Therefore, the efficient recovery of major components from spent LIBs is necessary for environmental protection and resource utilization [11,12].

A conventional LIB comprises a cathode, an anode, a separator, an electrolyte solution, current collectors (aluminum and copper foils), protective shells, and containers [13]. Currently, state-of-the-art technologies focus on recycling recycle-valuable metals, which are usually based on pyro-, hydro- and bio-metallurgical processes [14,15,16]. Organic binders wrapped on electrode particles are usually the main cause of difficulties in liberation and efficient extraction of electrode materials [17]. As reported, the main technologies used to achieve the liberation between the electrode material and aluminum/copper foils are mechanical crushing [16,17], ultrasonic cleaning [18], organic solvent dissolution [19,20], and high-temperature processes [21,22]. N-methyl-2- pyrrolidone (NMP) dissolution is usually used as a solvent to liberate electrode materials and foils, which is deemed as an effective and normal method of obtaining cathode material. Additionally, PVDF decomposes to lower molecule chain fluorinated organics such as vinylidene fluoride, 1,1,1,3,3,3-hexafluoro-propane, 1,2,4-trifluorobenzene, 1,3,5-trifluorobenzene and 1,4-difluorobenzene at 500 °C during the pyrolysis process [15]. Previous methods were found to remove the PVDF binder and liberate the electrode active materials from the substrate, but they failed to recover an organic binder to obtain reusable PVDF, although they have crucial drawbacks considering environmental concerns and workplace safety [23,24]. The formation of hydrofluoric acid and aromatic compounds during the decomposition of PVDF at high temperatures are the crucial bottlenecks to develop an industrial process because of their high corrosivity and hazardous nature, which is a significant threat to the atmosphere as well as workplace safety [25,26]. Additionally, chemicals used in organic solvent dissolution are toxic and highly dangerous for workers and the environment [27].

Based on the above issues, supercritical fluid (SCF) technology is a promising approach for removing and recycling organics and metals [28] and thus, has been investigated for implementation in the battery recycling process for both electrolyte, binder, and electrode recovery in the last decade [29,30,31,32]. The physical properties of the fluid change abruptly when it reaches the critical pressure and temperature, which leads to gas-like viscosity and liquid-like density. These features enable a SCF as an effective solvent that can dissolve the organics above the critical point and release it below [33,34,35]. It is important to choose a suitable solvent in consideration of economic and environmental issues. Carbon dioxide (CO_2_) is commonly preferred in supercritical fluid processing for organic and metal recovery due to several advantages such as its low cost, infinite cyclability, no secondary pollution, and its non-flammable properties compared to other solvents including water, ethanol, acetone, etc., as well as a relatively low supercritical point, 31 °C and 74 bar [36]. In LIB recycling, a few studies have investigated the implementation of SCF extraction for electrolyte recovery [37,38,39]. It is clear that SC-CO_2_ processing is an effective method to recover the electrolyte solvent due to the selectivity of the process for the organic components instead of polar inorganic materials. On the other hand, it is also possible to implement this process technology using chelating and modifying agents for metal recycling. Bertuol et al. [40] applied SC-CO_2_ with H_2_SO_4_ and H_2_O_2_ as a co-solvent to leach cobalt from lithium-ion batteries (LIBs). In their study, leaching tests were performed using SC-CO_2_ and co-solvents in comparison with conventional conditions. The use of supercritical conditions enables the extraction of more than 95 wt.% of the cobalt, with a minimized reaction time, and required a concentration of H_2_O_2_ compared to the conventional leaching at an atmospheric pressure. Zhang and Azimi [31] shared supporting results and showed that SC-CO_2_ extraction using tributylphosphate (TBP) as a complexing agent and HNO_3_ as a modifying agent, together with H_2_O_2_, improved the recovery of Li, Ni, Co and Mn compared to conventional leaching. Above all, SC-CO_2_ method can be applied to recover both organic and inorganic components of the LIB waste due to its superior and tunable properties. Although there is research on electrolyte and cathode material recovery using SC-CO_2_ technology, we recently validated the concept of PVDF binder solubility with the co-solvent assistant SC-CO_2_ [30].

In this study, SC-CO_2_ extraction was applied to enhance the dispersity of cathode material due to its superior solvent characteristics and the liberation of the cathode from aluminum foils was also obtained. Dimethyl sulfoxide (DMSO) can dissolve in both polar and non-polar solvents and thus, using it as a co-solvent promotes suitable physicochemical interactions between CO_2_ and PVDF. In comparison with traditional SC treatment, PVDF and cathode materials were recycled and retained the same characteristics. A scanning electron microscope (SEM), coupled with an energy dispersive spectrometer (EDS) and FT-IR, were applied to obtain the morphology and chemical composition of the surface elements of electrode material particles before and after SC-CO_2_ treatment. The effect of SC-CO_2_ parameters on the liberation of the cathode material was also investigated.

## 2. Results and Discussion

### 2.1. Characterization of Raw Cathode Material

**Figure 1:**
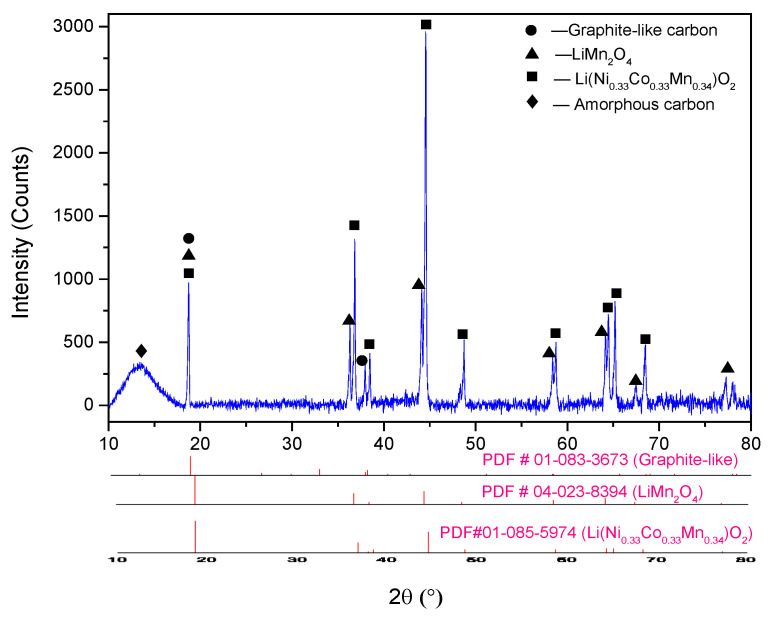
XRD pattern of raw cathode material.

The untreated cathode powder scraped from the cathode foils was characterized by ICP-OES and SEM-EDS. The elemental composition of the cathode active material was carried out by measuring the metals dissolved in aqua regia digestion (HCl: HNO_3_ = 3:1) through ICP-OES analysis, and the results are displayed in Table 1. The phase composition of the sample was analyzed with XRD (Figure 1), and the applied cathode material was found to be a mixture of LiNi_1/3_Co_1/3_Mn_1/3_O_2_, LiMn_2_O_4_, and carbon.

**Figure 2:**
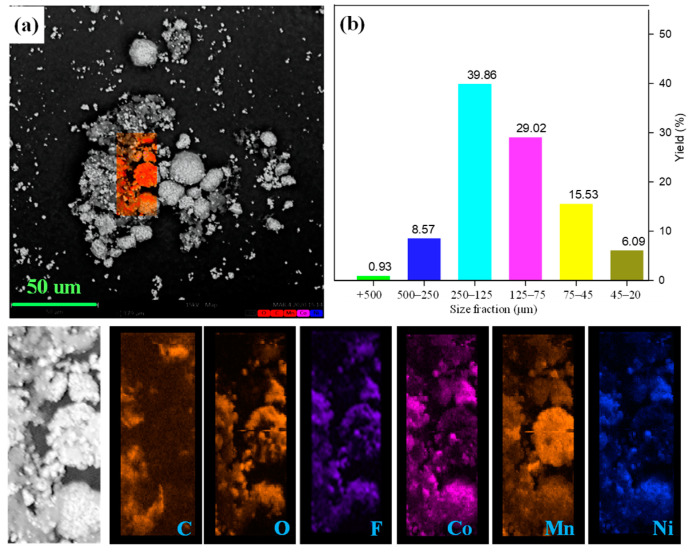
(**a**) SEM image together with EDS mapping and (**b**) size distribution of raw cathode material.

The raw cathode was analyzed with SEM and a sieving process to characterize its morphology and particle sizes. It is observed from Figure 2 that the single particles with a size of 10 to 30 μm are secondarily adhered by the binder in the Li-ion battery cathode pristine sample. Additionally, the surface of the cathode particles was covered with a flocculent layer. The distribution of fluorine and carbon elements can be used to characterize the occurrence of organic film as the main components of PVDF [37,38], indicating that the cathode particles are adhered together by PVDF. EDS was conducted to study the subsistent state of cathode particles and organic binders. As presented, metal elements (cobalt, nickel, and manganese) containing phase can be simultaneously detected in the single spherical and scattered particles, confirming the existence of LiNi_1/3_Co_1/3_Mn_1/3_O_2_ and LiMn_2_O_4_, which is confirmed by XRD analysis. In addition, the mapping of carbon and fluorine elements was highly enriched around the cathode materials that surrounded the gap. The sieving process also evaluated the size distribution of the cathode materials, and the results are shown in Figure 2b. In the sieving process, the cathode materials were divided into six fractions of −0.5 + 0.25 mm, −0.25 + 0.125 mm, −0.125 + 0.075 mm, −0.075 + 0.045 mm and −0.045 mm. The resulting present cathode material is mainly concentrated in −0.25 + 0.075 mm size fraction. In addition, the coarse fraction with +0.125 mm size accounts for 49.35% of the raw cathode material. Above all, the coarse powders and surrounded PVDF leads to the agglomeration between the particles and the existence of a strong binder that causes difficulties in easily liberating the cathode materials from the foils.

### 2.2. Removal of PVDF from Cathode Material by SC-CO_2_ Extraction

**Figure 3:**
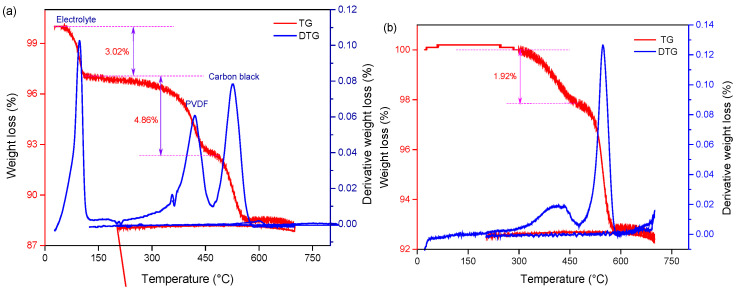
TG-DTG curve of (**a**) raw and (**b**) recovered cathode material treated by Sc-CO_2_ at 70 °C, 80 bar 13 min.

The cathode material was treated with an SC-CO_2_ extraction system (70 °C, 80 bar 13 min) to verify its feasibility. Thermogravimetric analyses (TGA) were performed to quantitatively determine the PVDF removal from spent cathode powder before and after SC-CO_2_ pre-treatment. Figure 3 shows the TGA results from the raw cathode material and the recovered material after cooling the DMSO solution process. The result of TG analysis is shown as weight loss and its derivative as a function of temperature. The characteristic peaks for the removal and decomposition of electrolytes and PVDF at 75–125 °C and 400–550 °C [39] can be observed in TGA curves, as shown in Figure 3a. After SC-CO_2_ pre-treatment, it is obvious that the peak of electrolyte disappeared and the intensity of the PVDF peak decreased remarkably, which indicates that organic binders and electrolytes were removed successfully. Meanwhile, the position and shape of the carbon black peak remain unchanged. It is concluded that the organic binder PVDF and electrolyte were removed from the cathode material. Thus, it is important to explore the appropriate conditions for PVDF removal and recovery of cathode material.

### 2.3. Transfer Behavior of PVDF Binder under SC-CO_2_ System

**Figure 4:**
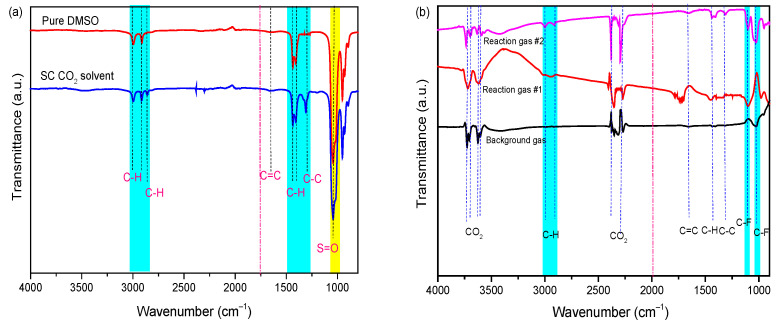
FT-IR spectra of (**a**) DMSO solvent [30] and (**b**) exhaust gas under Sc-CO_2_ at 70 °C, 80 bar 13 min.

According to the TGA results, the PVDF binder was removed from the cathode material, resulting in its dissolution in the DMSO solvent during the SC-CO_2_ extraction process. Therefore, it is also necessary to prove the binder separation by characterizing the dissolved PVDF in liquid samples that were collected from the reactor and the exhaust gas stream during the release of the pressure. Based on our previous studies [30], pure DMSO solvent, the collected solvent after treatment—which is dissolved PVDF in DMSO by SC-CO_2_ at 80 bar and 70 °C for 13 min—and exhaust gas were characterized by FTIR, as shown in Figure 4a. The peaks of the DMSO spectrum (cyan strip) at 3010 cm^−1^ and 2915 cm^−1^ are attributed to the anti-symmetrical and symmetrical stretching vibration of methyl C-H bond, and the peak at 1440 cm^−1^ and 1405 cm^−1^ belongs to methyl C-H bending vibration, respectively. Additionally, the peak at 1057 cm^−1^ belongs to the S=O bond. In the spectra of solvent and exhaust gas, the peaks at 1103 cm^−1^ and 1022 cm^−1^ belong to the anti-symmetrical and symmetrical stretching vibration of the C-F bond, which is attributed to PVDF. After being treated in the SC-CO_2_-DMSO fluid process, the peaks of the C-H/C=C appeared in liquid FT-IR spectra due to the dissolution of PVDF in the DMSO solution, and then were transferred to the gas efflux component. Based on the above analysis, the FTIR results indicate that it is feasible to dissolve PVDF binder from a cathode mixture with DMSO solvent under the SC-CO_2_ process. However, traces of the DMSO solvent were detected in the exhaust gas due to its transportation through the high-pressure CO_2_ stream.

### 2.4. Effect of SC-CO_2_ Treatment on the Liberation of Cathode Material

#### 2.4.1. Effect of SC-CO_2_ Treatment on the Size Distribution of Cathode Material

**Figure 5:**
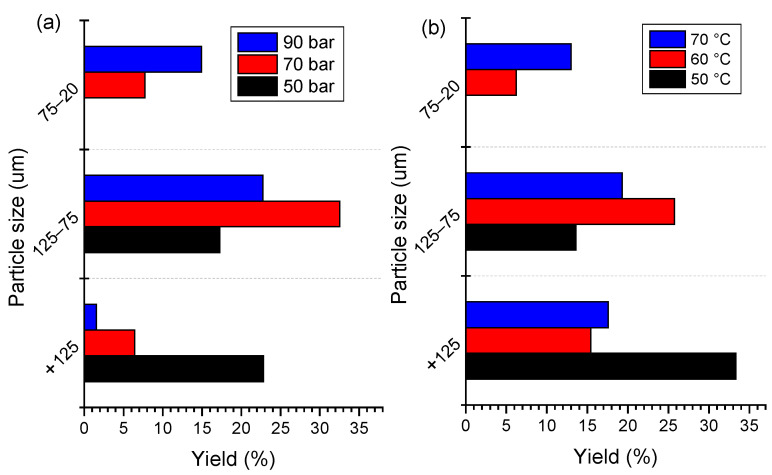
Size distribution of SC-CO_2_ product under different (**a**) pressures at 70 °C and (**b**) temperatures at 80 bar for 13 min.

The effects of SC-CO_2_ extraction conditions on the liberation of the cathode materials were evaluated by their size distribution. The size distribution of extraction products under different pressures and temperatures are shown in Figure 5. The fine fraction with −75 μm size accounts for 21.63% of the raw cathode material. The higher content of a coarse size fraction demonstrates that a high number of cathode materials cannot be liberated from foils due to agglomeration between the particles and the binder. After SC-CO_2_ treatment, the coarse size fraction (+75 μm) decreases accordingly with the increase in pressure and temperature. It is clear that the −20 μm fraction of the cathode material under 70 bar and 90 bar accounts for 8.21% and 14.77%, receptively, while this size is absent in the raw cathode material. Meanwhile, the content of the finer size fraction increased with the increase in temperature. As shown in Figure 5b, the content of −125 + 75 μm fraction accounts for 13.26% under 50 °C, while it decreased to 27.64% when the temperature increased to 60 °C. the −20 μm fraction increased obviously with the increase in pressure. The size distribution denotes that the removal of PVDF by SC-CO_2_ with the co-solvent method is useful for cathode material liberation. Based on the above size distribution analysis, the SC-CO_2_-DMSO fluid system is confirmed to be effective for de-agglomeration and liberation of cathode material. In comparison with the other techniques, a lower temperature and time are needed during the SC-CO_2_ process, which is found to be a low energy consumption process to remove the organic binder from spent LIBs.

#### 2.4.2. Optimization on the Liberation of Cathode Material

**Figure 6:**
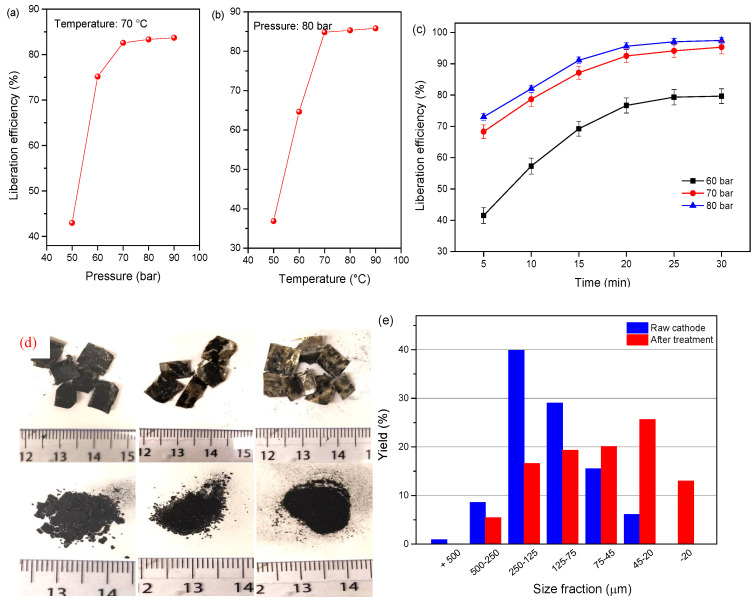
Liberation efficiency of cathode material as a function of (**a**) pressure at 70 °C for 13 min, (**b**) temperature at 80 bar for 13 min, and (**c**) liberation as a function of time and pressure at 70 °C, (**d**) picture of the samples, (**e**) size distribution of the cathode powders before and after treatment at 80 bar, 20 min, and 70 °C.

**Figure 7:**
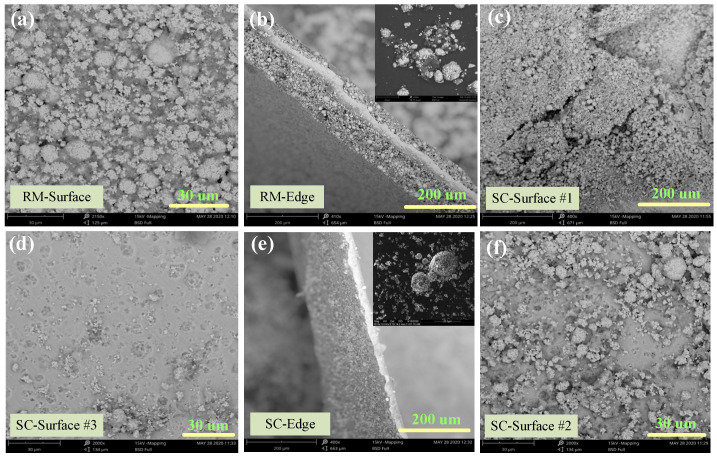
De-agglomeration and dispersibility characteristics analyzed by SEM for (**a**), (**b**) raw material, (**c**,**f**) 80 bar, 13 min, and 70 °C and (**d**,**e**) 80 bar, 20 min, and 70 °C.

Temperature and pressure affect the fluid property of SC-CO_2_ and thus, the dissolution of the organic binder change abruptly. The liberation efficiency of the cathode materials at the single-factor test of different pressures (50–90 bar) and temperatures (50–90 °C) for 13 min is shown in Figure 6a. As it can be observed, the liberation efficiency of cathode powders remarkably increased by pressurizing the reactor from 50 to 70 bar, but the liberation efficiency approached the stabilization at pressures over 70 bar. In the case of the temperature effect, the liberation efficiency presents a sharp increase with the elevating temperature from 50 to 70 °C, and then the trend almost stabilized. The results indicate an effective liberation efficiency when the temperature and pressure exceed the critical point, which is attributed to the dissolution of PVDF by co-solvent DMSO. The relationships between the operational parameters were presented by plotting two independent variables with the liberation efficiency. Figure 6b shows the variation regulation of the liberation efficiency under the effects of two independent variables of temperature and pressure. It is obvious that the liberation efficiency of cathode material under 50 bar is lower than the pressure increase to 70 and 90 bar. It is concluded that the optimal liberation efficiency of 96.7% for the cathode material is obtained under 80 bar, 20 min, and 70 °C. The size distribution of the raw and treated cathode material indicates that the finer size fraction of −45 μm increased from 6.1% to 39.61%, and the liberation effect is improved as shown in Figure 7c,d.

#### 2.4.3. Morphology Liberation from Al Foil, between Particles

SEM analysis of the cathode material before and after Sc-CO_2_ treatment was conducted to investigate the morphological changes, and the results are shown in Figure 7. Apparent agglomeration with several secondary particles adhered by the binder in the Li-ion battery cathode, pristine sample (Figure 7a). The SEM image (Figure 7b) of the edge face for the pristine sample indicates that the particles adhere tightly to Al foil. With the increase in temperature and pressure of SC fluid, the cathode powders gradually liberated from Al foil. At first, cracks appear on the surface of the cathode foil which is attributed to the PVDF binder dissolution in the DMSO solvent as shown in Figure 7c. Then, the particles gradually escape from the foil and the potholes appear on the foil surface as presented in Figure 7c,f. The illustration of Figure 7e shows that the mutual liberation of the cathode material is improved after SC-CO_2_ treatment. It is obvious that the degree of aggregation decreased significantly, owing to the removal of the organic binder, and the cathode particles are dispersed into a smaller fragment, which facilitates the liberation efficiency of the cathode material from the cathode foil. To sum up, the cathode material and current foils were recycled separately under SC-CO_2_ treatment with high efficiency.

### 2.5. Proposed Mechanism of SC-CO_2_ Extraction of Cathode Material

**Figure 8:**
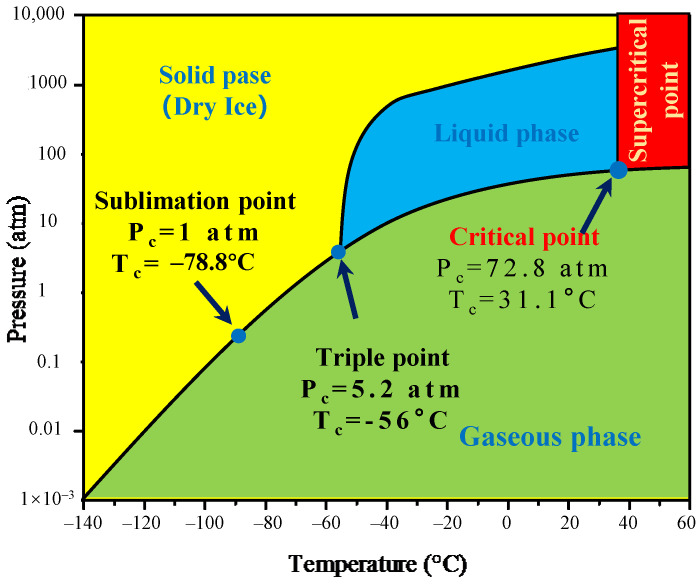
Supercritical CO_2_ phase diagram.

The de-agglomeration and recovery of cathode materials from spent lithium-ion batteries were conducted using supercritical carbon dioxide extraction, and the flow chart is presented in Figure 8. Since PVDF is polar, it is not expected to dissolve in non-polar CO_2_, due to limited interactions between the molecules at a relatively low pressure and temperature. However, DMSO can dissolve in both polar and non-polar solvents and thus, using it as a co-solvent promotes suitable physicochemical interactions between CO_2_ and PVDF. The improved molecular interaction with SC-CO_2_ leads to the decreasing free volume difference between the solvent and the polymer, which facilitates the dissolution of the polymer compared with the traditional liquid phase environment as shown in Figure 8.

**Figure 9:**
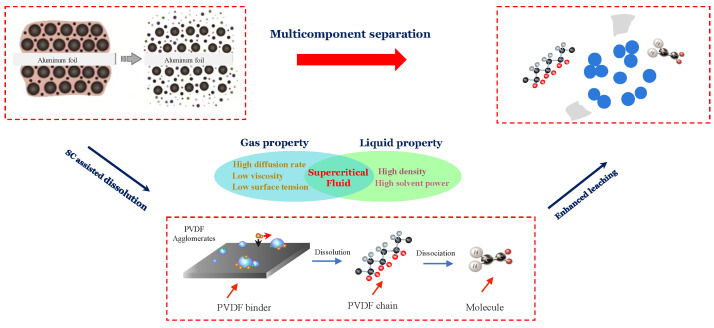
Flow chart of SC-CO_2_ extraction of cathode material.

Additionally, PVDF has a -[CH_2_-CF_2_]_n_- structure and fluorine (F) in the molecule has a high tendency to gain electron and bonding c capability because it is the most electronegative element [41]. Studies on the dissolution of fluorinated polymers by SC-CO_2_ shows the weak interaction and formation of the C-F dipole [42,43]. Thus, it is suggested that the F atoms in the chain attract the CO_2_ and form a weak Lewis acid (LA)–base (LB) pair. Additionally, C-H bonds in the fluorinated hydrocarbons can interact with CO_2_ oxygen atoms, leading to the formation of weak collaborative hydrogen bonding (C-H⋯O) [43]. These two specific interactions enhance the dissolution of PVDF in CO_2_. Therefore, we proposed the flow chart of SC-CO_2_ extraction of cathode material as shown in Figure 9. It should be highlighted that the role of DMSO as a co-solvent is critical to activate the molecular interactions between the solvent and the polymer. Therefore, the proposed SC-CO_2_ and co-solvent system effectively separate the PVDF from Li-ion battery electrodes. This recycling approach is a promising candidate for an alternative pre-treatment method due to its high efficiency, relatively low energy consumption, and other features.

The experimental materials used in this research are only carbon dioxide (CO_2_) and DMSO to remove the PVDF binder and liberate the cathode material. CO_2_ is deemed as a good solvent due to its many advantages, including low cost and infinite cyclability, compared to other solvents. Considering the high degree of liberation of the electrode active materials, this approach can facilitate the direct recycling of the cathode materials, as well as ease the hydrometallurgical treatment. Since the metals derived from cathode materials, including Li, Co, Ni, etc., are critical and strategic raw materials it is important to develop a pre-treatment approach to increase their recyclability. In addition, the application of SC-CO_2_ can decrease the dosage of an organic solvent and be beneficial for reducing their manufacturing-related energy consumption. Therefore, considering the environmental and economic prospects, the proposed process is meaningful to implement in the spent LIBs recycling field.

## 3. Method

### 3.1. Materials and Reagents

The experimental samples of spent aluminum laminated batteries (ALBs) used in this study were provided by a vehicle Swedish manufacturer. These batteries were firstly discharged in an electrolyte solution (10% NaCl, *w*/*v*) for about 24 h to prevent possible accidents such as short-circuiting and self-ignition during the dismantling stage. Then, they were manually dismantled into different components including cases, separators, anode, and cathode foils. The obtained cathode foils were cut into around 1 × 0.6 cm^2^ pieces and used for the subsequent extraction process. The reagent DMSO (analytically pure) was purchased from Sigma-Aldrich, Ltd., St. Louis, MO, USA, and its nontoxicity was identified from the Material Safety Data Sheet (MSDS). Pure CO_2_ gas (purity 99.998%) was used for extraction experiments. The abbreviations involved in this article are shown in Table 2.

### 3.2. Experimental Procedures

**Figure 10:**
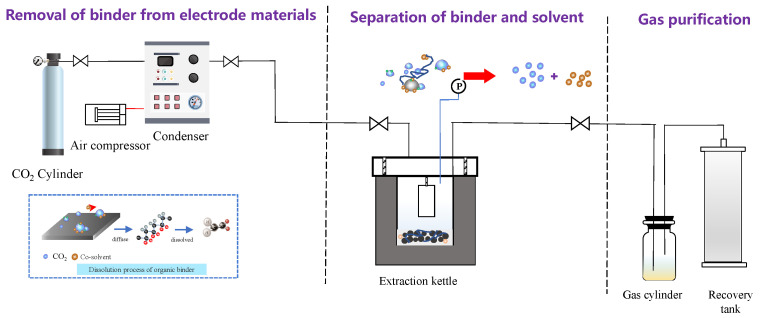
Self-developed SC-CO_2_ processing system illustration.

The supercritical conditions were prepared using an ISCO syringe pump (Teledyne ISCO model 260D, Lincoln, NE, USA) with a series D pump controller. The experimental instrument is shown in Figure 10. The supercritical fluid apparatus used for extraction of spent LIBs was constructed inside of the fume hood. The whole system, including the reactor and the syringe pump, is heated via a thermostat water bath to keep a stable temperature range and to avoid freezing during pressure reliefs. Before the experiments, samples were placed in a reactor constructed of 316 stainless steel and were fixed with an iron pedestal. For each experiment, the supercritical extractions were performed at a given temperature and CO_2_ pressure. A total of 0.3 g (1 × 1 cm^2^) solid cathode foils were added into the reactor that contained 4 mL of co-solvent DMSO solution. To obtain the optimum conditions, various pressures (50–90 bar), temperatures (50–90 °C), and duration periods (5–30 min) were tested during the SC-CO_2_ extraction process. After being treated by SC-CO_2_, the extraction vessel was removed, and the products that contained solvent, Al foils, and cathode materials were directly collected. Liquid samples were analyzed by ATR-FTIR after cooling to room temperature. The residual solid powder samples were filtered from the slurry and characterized by following electron microscopy and sieving analysis. The experiments were triplicated and the results were obtained from the mean values of three independent tests.

### 3.3. Measurement and Characterization

The morphologies and surficial elemental distribution of the electrode materials were analyzed using a scanning electron microscope (SEM-EDS, Phenom ProX, Phenom, Ambler, PA, USA) in backscattered electrons mode. The inorganic components of raw samples and the leaching residues were characterized by X-ray diffraction (XRD, D8 ADVANCE, Bruker, Germany) employing Cu Kα radiation (35 KV and 30 mA). Samples were scanned from 5° to 90° with a 0.02° step size and 0.2 s/step scan speed. The thermogravimetric analyzer (TGA, Q500, TA) with N_2_ running gas (10 °/min heating rate) was used to determine and compare the thermochemical properties of the raw PVDF and SC-CO_2_ recovered PVDF materials. Metal concentrations in the solution were determined by inductively coupled plasma optical emission spectrometry (ICP-OES, iCAP 6000 series, Thermo Scientific, Waltham, MA, USA). Pure DMSO and PVDF remains were characterized using a Perkin Elmer Spectrum Two FTIR spectrometer with a monolithic diamond ATR accessory (PerkinElmer, UATR two, Waltham, MA, USA). The recovery yield of the cathode material defined by Formula (1) was adopted to evaluate the recovery effect.

(1) $$R=\frac{m_{0}-m_{t}}{m_{0}-m_{f}}\times 100\;\;$$

where *R* is the recovery rate (%), *m_t_* is the weight of the remained cathode material and Al foil after the SC-CO_2_ dissolution process, *m*_0_ is the total weight of the cathode foil before treatment, and m_f_ is the weight of clean Al foil.

A total of 0.3 g (1 × 1 cm^2^) solid cathode foils were used as the raw material, and the recovery rate was calculated based on the weight of each component. In order to accurately calculate the mass of the aluminum foil and electrode materials after dissociation, small slices of electrode materials of different sizes were measured, as shown in Table S1. Therefore, linear fitting was performed on the relationship between *m*_0_ and m_f_, that is y = 0.1296x + 0.0008.

## 4. Conclusions

In this study, SC-CO_2_, combined with a co-solvent dimethyl sulfoxide (DMSO), was adapted to facilitate the liberation of cathode material from spent LIBs cathode material and aluminum foils. The experimental results indicate that the optimal liberation efficiency was obtained using a supercritical CO_2_-DMSO system under the optimum conditions of 70 °C temperature, 80 bar pressure, and 20 min process time. The FTIR and TG characterization results revealed that polyvinylidene fluoride was dissolved in the DMSO solvent and thus, were removed from the cathode material, which is beneficial for the complete recycling of organics recycled from spent LIBs. Further, the liberation effect between the cathode material and the aluminum foil was conducted and analyzed by SEM and size distribution. SEM characterization revealed that the degree of aggregation decreased significantly, owing to the removal of the organic binder, and the cathode particles were dispersed into smaller fragments. Meanwhile, the size distribution analysis of the SC-CO_2_ product also demonstrates that the finer size fraction of the cathode material is improved, which facilitates the intergranular liberation of the cathode powders. In comparison with traditional solvent-treated methods, DMSO can dissolve in both polar and non-polar solvents and thus, using it as a co-solvent promotes suitable physicochemical interactions between CO_2_ and PVDF. The proposed SC-CO_2_ and co-solvent system effectively separates the PVDF from Li-ion battery electrodes. The suggested process is found to be effective and sustainable in recycling organic binders and liberating cathode material from spent LIBs.

## Figures and Tables

**Table 1:** Elemental composition of cathode active material determined by ICP-OES.

Elements	Li	Co	Ni	Mn	Al
Content (wt.%)	5.91	11.7	11.5	26.02	0.06

**Table 2:** The abbreviations involved in this article.

Abbreviations	Explanation
SC	Supercritical
SCF	Supercritical fluid
EV	Electric vehicles
LIB	Lithium-ion battery
PVDF	Polyvinylidene difluoride
DMSO	Dimethyl sulfoxide
SEM	Scanning electron microscope
EDS	Energy dispersive spectrometer
FTIR	Fourier Transform Infrared Spectrometer

## Data Availability

Data are contained within the article and Supplementary Materials.

## Supplementary Materials

The following supporting information can be downloaded at: https://www.mdpi.com/article/10.3390/molecules29071638/s1. Table S1. The relationship between *m*_0_ (cathode + Al weight) and mf (Al foil).

## References

[1] Zhang X., Bian Y., Xu S., Fan E., Xue Q., Guan Y., Wu F., Li L., Chen R. (2018). Innovative application of acid leaching to regenerate Li (Ni_1/3_Co_1/3_Mn_1/3_)O_2_ cathodes from spent lithium-ion batteries. ACS Sustain. Chem. Eng..

[2] Fu Y., He Y., Qu L., Feng Y., Li J., Liu J., Zhang G., Xie W. (2019). Enhancement in leaching process of lithium and cobalt from spent lithium-ion batteries using benzenesulfonic acid system. Waste Manag..

[3] Wu Q., Jiang R., Mu L., Xu S. (2018). Fe_3_O_4_ anodes for lithium batteries: Production techniques and general applications. Comptes Rendus Chim..

[4] Fu Y., He Y., Yang Y., Qu L., Li J., Zhou R. (2020). Microwave reduction enhanced leaching of valuable metals from spent lithium-ion batteries. J. Alloys Compd..

[5] Fu Y., He Y., Chen H., Ye C., Lu Q., Li R., Xie W., Wang J. (2019). Effective leaching and extraction of valuable metals from electrode material of spent lithium-ion batteries using mixed organic acids leachant. J. Ind. Eng. Chem..

[6] (2019). Projected Lithium Ion Battery Market Size Worldwide in 2017 and 2025. https://www-statista-com.proxy.lib.chalmers.se/statistics/235316/global-lithium-battery-market/.

[7] Zhang Y., Meng Q., Dong P., Duan J., Lin Y. (2018). Use of grape seed as reductant for leaching of cobalt from spent lithium-ion batteries. J. Ind. Eng. Chem..

[8] Zheng X., Gao W., Zhang X., He M., Lin X., Cao H., Zhang Y., Sun Z. (2016). Spent lithium-ion battery recycling—Reductive ammonia leaching of metals from cathode scrap by sodium sulphite. Waste Manag..

[9] Meng Q., Zhang Y., Dong P., Liang F. (2018). A novel process for leaching of metals from LiNi_1/3_Co_1/3_Mn_1/3_O_2_ material of spent lithium ion batteries: Process optimization and kinetics aspects. J. Ind. Eng. Chem..

[10] Shi Y., Chen G., Chen Z. (2018). Effective regeneration of LiCoO_2_ from spent lithium-ion batteries: A direct approach towards high-performance active particles. Green Chem..

[11] Zhang X., Li L., Fan E., Xue Q., Bian Y., Wu F., Chen R. (2018). Toward sustainable and systematic recycling of spent rechargeable batteries. Chem. Soc. Rev..

[12] Wang M., Tan Q., Chiang J.F., Li J. (2017). Recovery of rare and precious metals from urban mines—A review. Front. Environ. Sci. Eng..

[13] Zhang G., He Y., Feng Y., Wang H., Zhang T., Xie W., Zhu X. (2018). Enhancement in liberation of electrode materials derived from spent lithium-ion battery by pyrolysis. J. Clean. Prod..

[14] Yaug L., Xi G., Xi Y. (2015). Recovery of Co, Mn, Ni, and Li from spent lithium ion batteries for the preparation of LiNixCoyMnzO_2_ cathode materials. Ceram. Int..

[15] Golmohammadzadeh R., Rashchi F., Vahidi E. (2017). Recovery of lithium and cobalt from spent lithium-ion batteries using organic acids: Process optimization and kinetic aspects. Waste Manag..

[16] Chen X., Kang D., Cao L., Li J., Zhou T., Ma H. (2019). Separation and recovery of valuable metals from spent lithium ion batteries: Simultaneous recovery of Li and Co in a single step. Sep. Purif. Technol..

[17] Zhang G., He Y., Feng Y., Wang H., Zhu X. (2018). Pyrolysis-Ultrasonic-Assisted Flotation Technology for Recovering Graphite and LiCoO_2_ from Spent Lithium-Ion Batteries. ACS Sustain. Chem. Eng..

[18] Ku H., Jung Y., Jo M., Park S., Kim S., Yang D., Rhee K., An E.-M., Sohn J., Kwon K. (2016). Recycling of spent lithium-ion battery cathode materials by ammoniacal leaching. J. Hazard. Mater..

[19] Zhang T., He Y., Ge L., Fu R., Zhang X., Huang Y. (2013). Characteristics of wet and dry crushing methods in the recycling process of spent lithium-ion batteries. J. Power Sources.

[20] Li J., Shi P., Wang Z., Chen Y., Chang C.-C. (2009). A combined recovery process of metals in spent lithium-ion batteries. Chemosphere.

[21] Xin Y., Guo X., Chen S., Wang J., Wu F., Xin B. (2016). Bioleaching of valuable metals Li, Co, Ni and Mn from spent electric vehicle Li-ion batteries for the purpose of recovery. J. Clean. Prod..

[22] Nayaka G.P., Pai K.V., Manjanna J., Keny S.J. (2016). Use of mild organic acid reagents to recover the Co and Li from spent Li-ion batteries. Waste Manag..

[23] Hao J., Wang H., Chen S., Cai B., Ge L., Xia W. (2014). Pyrolysis characteristics of the mixture of printed circuit board scraps and coal powder. Waste Manag..

[24] Choi S.-S., Kim Y.-K. (2012). Microstructural analysis of poly(vinylidene fluoride) using benzene derivative pyrolysis products. J. Anal. Appl. Pyrolysis.

[25] Lv W., Wang Z., Cao H., Sun Y., Zhang Y., Sun Z.H. (2018). A Critical Review and Analysis on the Recycling of Spent Lithium-Ion Batteries. ACS Sustain. Chem. Eng..

[26] Yao Y., Zhu M., Zhao Z., Tong B., Fan Y., Hua Z. (2018). Hydrometallurgical Processes for Recycling Spent Lithium-Ion Batteries: A Critical Review. ACS Sustain. Chem. Eng..

[27] Lombardo G., Ebin B., Foreman MR S.J., Steenari B.M., Petranikova M. (2020). Incineration of EV Lithium-ion batteries as a pretreatment for recycling–determination of the potential formation of hazardous by-products and effects on metal compounds. J. Hazard. Mater..

[28] Goto M. (2009). Chemical recycling of plastics using sub- and supercritical fluids. J. Supercrit. Fluids.

[29] Grützke M., Mönnighoff X., Horsthemke F., Kraft V., Winter M., Nowak S. (2015). Extraction of lithium-ion battery electrolytes with liquid and supercritical carbon dioxide and additional solvents. RSC Adv..

[30] Fu Y., Schuster J., Petranikova M., Ebin B. (2021). Innovative recycling of organic binders from electric vehicle lithium-ion batteries by supercritical carbon dioxide extraction. Resour. Conserv. Recycl..

[31] Zhang J., Azimi G. (2022). Recycling of lithium, cobalt, nickel, and manganese from end-of-life lithium-ion battery of an electric vehicle using supercritical carbon dioxide. Resour. Conserv. Recycl..

[32] Schwich L., Schubert T., Friedrich B. (2021). Early-stage recovery of lithium from tailored thermal conditioned black mass part I: Mobilizing lithium via supercritical CO_2_-carbonation. Metals.

[33] Erkey C. (2000). Supercritical carbon dioxide extraction of metals from aqueous solutions: A review. J. Supercrit. Fluids.

[34] Nalawade S.P., Picchioni F., Janssen L. (2006). Supercritical carbon dioxide as a green solvent for processing polymer melts: Processing aspects and applications. Prog. Polym. Sci..

[35] Tutek K., Masek A., Kosmalska A., Cichosz S. (2021). Application of Fluids in Supercritical Conditions in the Polymer Industry. Polymers.

[36] Herrero M., Mendiola J.A., Cifuentes A., Ibáñez E. (2010). Supercritical fluid extraction: Recent advances and applications. J. Chromatogr. A.

[37] Nowak S., Winter M. (2017). The Role of Sub- and Supercritical CO_2_ as “Processing Solvent” for the Recycling and Sample Preparation of Lithium Ion Battery Electrolytes. Molecules.

[38] Grützke M., Kraft V., Weber W., Wendt C., Friesen A., Klamor S., Winter M., Nowak S. (2014). Supercritical carbon dioxide extraction of lithium-ion battery electrolytes. J. Supercrit. Fluids.

[39] Mu D., Liang J., Zhang J., Wang Y., Jin S., Dai C. (2022). Exfoliation of Active Materials Synchronized with Electrolyte Extraction from Spent Lithium-Ion Batteries by Supercritical CO_2_. ChemistrySelect.

[40] Bertuol D.A., Machado C.M., Silva M.L., Calgaro C.O., Dotto G.L., Tanabe E.H. (2016). Recovery of cobalt from spent lithium-ion batteries using supercritical carbon dioxide extraction. Waste Manag..

[41] Yu J., He Y., Li H., Xie W., Zhang T. (2017). Effect of the secondary product of semi-solid phase Fenton on the flotability of electrode material from spent lithium-ion battery. Powder Technol..

[42] Zhang T., He Y., Wang F., Li H., Duan C., Wu C. (2014). Surface analysis of cobalt-enriched crushed products of spent lithium-ion batteries by X-ray photoelectron spectroscopy. Sep. Purif. Technol..

[43] Zhan R., Payne T., Leftwich T., Perrine K., Pan L. (2020). De-agglomeration of cathode composites for direct recycling of Li-ion batteries. Waste Manag..

