# Supplementary material for: Resource Recovery of Spent Lithium-Ion Battery Cathode Materials by a Supercritical Carbon Dioxide System
Source: Molecules. 2024 Apr 5;29(7):1638. doi: 10.3390/molecules29071638 (PMC11013235; doi:10.3390/molecules29071638)
Supplement: Supplementary file 1 [file molecules-29-01638-s001.zip › molecules-2901872-supplementary.pdf]

Table S1 The relationship between m_0 (cathode + Al weight) and m_f (Al foil)

Size (cm^2)	m_0 (g)	m_f (g)
1.1 ×0.6	0.0328	0.0047
1.2×0.8	0.0393	0.0054
1-1.1×1.2	0.0518	0.0083
2.55×2.6	0.2658	0.0352
3.1-3.4×3.2-3.9	0.3523	0.0464
